# A Case Report of Glomus Tympanicum Complicated With Facial Nerve Palsy 

**DOI:** 10.22038/IJORL.2022.64737.3217

**Published:** 2022-11

**Authors:** Christodoulos Dimakis, Despoina Beka, Eustratios Papageorgiou, Nikolaos Tsetsos, Alexandros Poutoglidis, Athanasia Gortsali, Alexandros Nomikos, Georgios Karatzias

**Affiliations:** 1 *Department of Otorhinolaryngology-Head and Neck Surgery, * *General Hospital Asklip* *ι* *eio Voula, Athens, Greece.*; 2 *Department of Otorhinolaryngology-Head and Neck Surgery, G.Papanikolaou* *General Hospital, Thessaloniki, Greece.*; 3 *Department of Pathology, General Hospital Asklip* *ι* *eio Voula, Athens, Greece.*

**Keywords:** Facial nerve paralysis, Glomus tympanicum, Paraganglioma

## Abstract

**Introduction:**

Generally, glomus tumors are considered tumors of the autonomic system arising from chromaffin cells of the parasympathetic paraganglia of the skull base and neck. Glomus tympanicum is the most common primary tumor of the middle ear cavity and it arises from the paraganglia of the middle ear.

**Case Report:**

We present a case of glomus tympanicum presented in a 70-year-old woman, complicated with facial nerve palsy which at first sight was misdiagnosed as cholesteatoma. Patient presented in our clinic because of otorrhea, pulsatile tinnitus and hearing loss in the right ear. However, facial nerve function was good in the first examination (40 days before the surgery). Eventually, she treated successfully with a canal wall down mastoidectomy. Technique had been chosen because of the mass size and the involvement of external auditory canal, after a discussion with the patient.

**Conclusions:**

Although histologically benign, glomus tympanicum is slow growing and destructs adjacent tissues potentially. The two most common complaints are hearing loss (conductive) and pulsatile tinnitus. These neoplasms are more common in women and they can be diagnosed by CT or MRI scan. It is of high importance physicians suspect a glomus tumor when patient ‘s clinical findings are hearing loss and pulsatile tinnitus and use an intravascular agent in imaging so that the differential diagnosis will be supported.

## Introduction

Glomus tympanicum is the most common primary tumour of the middle ear cavity (1) therefore its study it is of great interest in the area of otology and oncology. Although histologically benign, glomus tympanicum is slow growing and destructs adjacent tissues potentially (2). The two most common complaints are hearing loss (conductive) and pulsatile tinnitus (3). We present a case of glomus tympanicum presented in a 70-year-old woman, complicated with facial nerve palsy, that has been treated successfully with a canal wall down mastoidectomy. Its size, pathologic findings, difficulties in diagnosis and management strategy applied for its treatment, highlight the importance of its publication.

## Case Report

**Fig 1:**
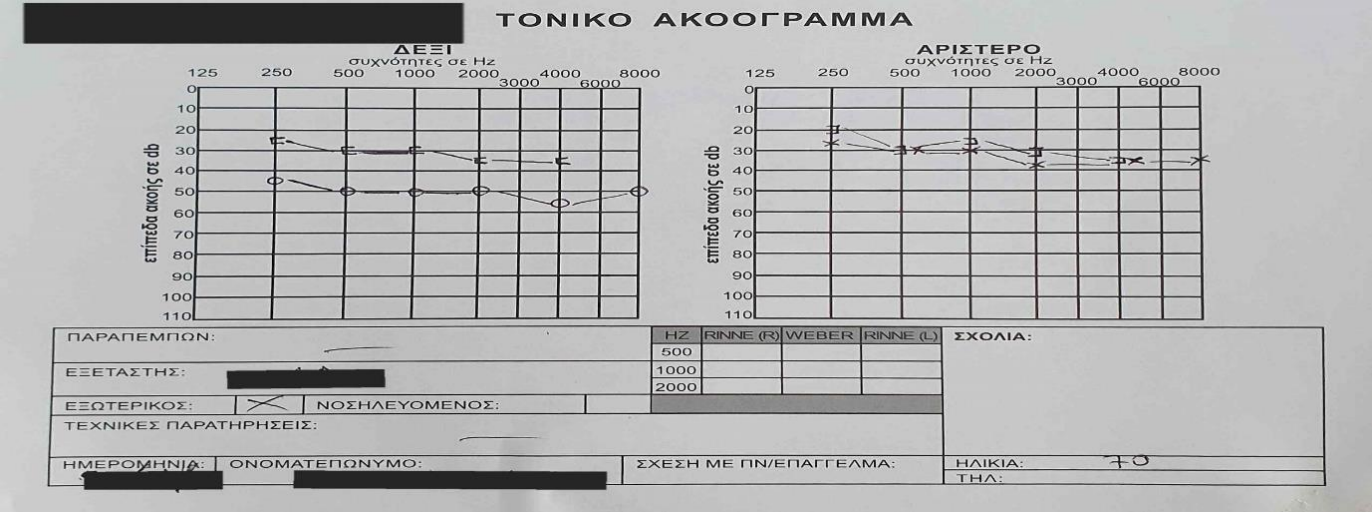
Pure tone audiometry. There is moderate mixed hearing loss in right ear and mild sensorineural hearing loss in left ear

 A 70-year-old woman presented at our Ear, Nose, and Throat (ENT) clinic complaining about bad smell and otorrhea, pulsatile tinnitus and sense of ear fullness on the right side over the last 12 months. Her past medical history was otherwise normal. On otomicroscopy, there was identified a mass occupying completely the external auditory canal, which rendered impossible the overview of the tympanic membrane. Moreover, there was a mild and painless mastoid redness respectively. A pure tone audiometry was performed and it revealed a moderate mixed hearing loss in right ear and a mild sensorineural hearing loss in left ear (Figure 1). 

**Fig 2:**
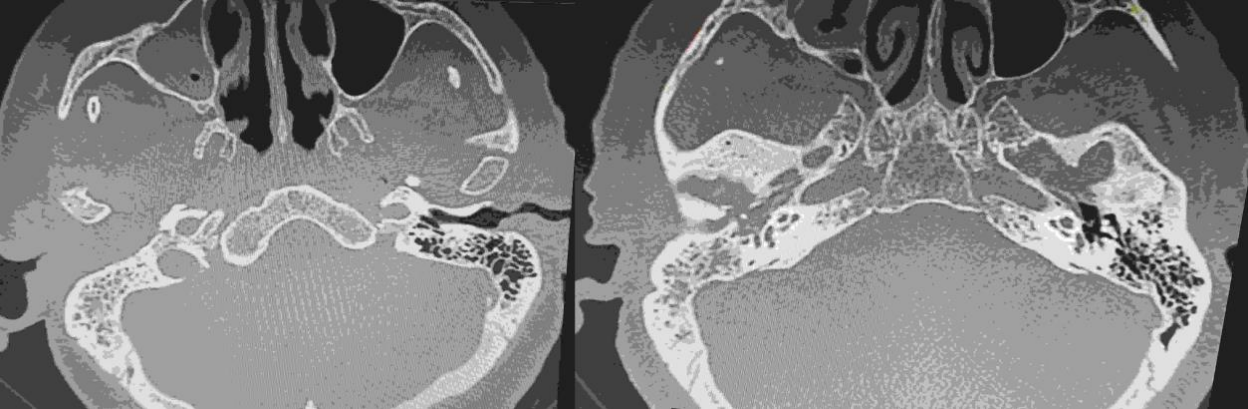
Computed tomography of temporal boned shows soft tissue density in the whole middle ear cavity with expansion in the mastoid and the external auditory canal (EAC), soft tissue ossification elements and smooth erosions of petrous part of temporal bone

A computed tomography (CT) was scheduled revealing soft tissue density in the whole middle ear cavity with expansion in the mastoid and the external auditory canal (EAC), soft tissue ossification elements and smooth erosions of petrous part of temporal bone (Figure 2). 

**Fig 3:**
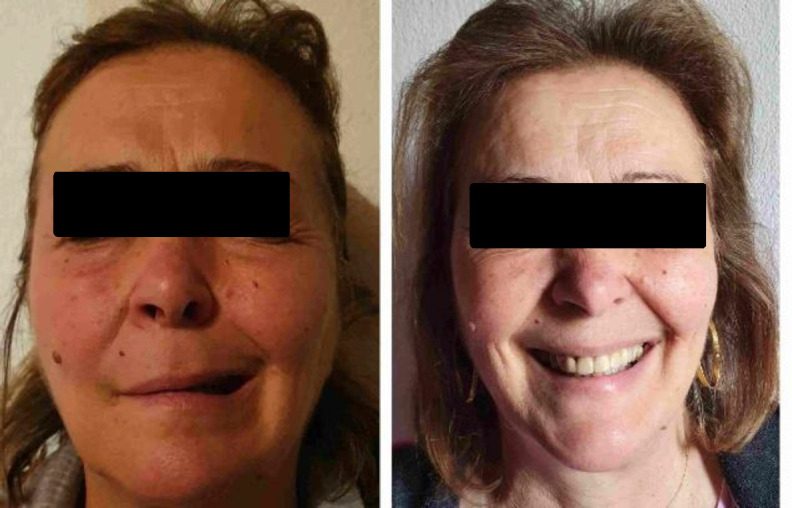
Facial nerve palsy (House-Brackmann III) preoperatively and facial nerve function improvement postoperatively (House-Brackmann I)

Radiology report attributed these findings to middle ear cholesteatoma. The patient was prescribed local antibiotics with the aim to resolve otorrhea. Because of the excessive mass size, which occupied the whole middle ear and seemed to had affected the bone structure in CT scan and because our team wanted to be certain about the complete exclusion of the tumor, a radical mastoidectomy was planned one month later. The night before the surgery the patient presented facial nerve palsy (House-Brackmann III) (Figure 3). 

**Fig 4:**
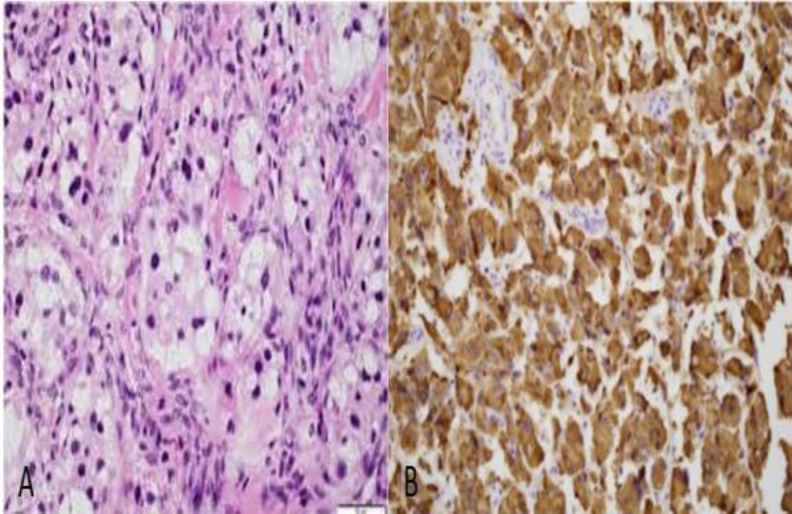
Histopathological examination. A, Neoplastic cells arranged in distinctive clusters separated by fibrovascular stroma. B, CD56x200 strong and diffuse expression

There was administrated intravenous steroid (methylprednisolone 500 mg) immediately. The following day, at the beginning of the surgery, fragments of the tumor of the EAC were biopsied. Biopsy caused continuous bleeding and the attempt for hemostasis doubled the surgical time. Eardrum perforation and ossicular erosions (malleus and incus were fully destroyed, stapes was partly eroded) had been found during the operation. Frozen section reported possible malignancy with concomitant inflammatory elements, without covering epithelium. Αn uncomplicated canal wall down mastoidectomy was performed. Facial nerve palsy resolved postoperatively (House-Brackmann I) (Figure 3). The final histopathologic examination was indicative of a Fisch type B paraganglioma. The Fisch classification of glomus tumors is based on extension of the tumor to surrounding anatomic structures and is closely related to mortality and morbidity. In type a tumor is limited in middle ear, in type B tumor is restricted to tympanomastoid site, in type C tumor involves the infra-labyrinth portion towards the petrous apex and in type D there is intracranial invasion. Histopathologist found neoplastic cells arranged in distinctive clusters separated by fibrovascular stroma and CD56 expressed strong and diffuse (Figure 4). 

**Fig 5:**
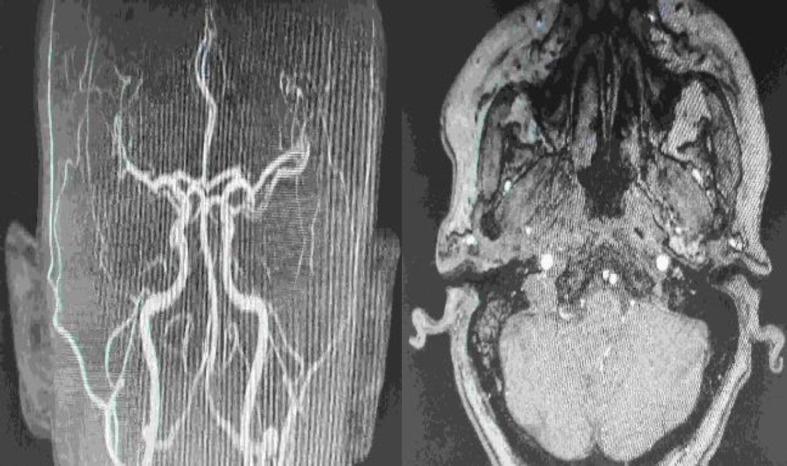
Post-operative magnetic resonance angiography with no evidence of disease in the vascular system. It also shows the presence of chronic inflammatory lesions in petrous part of temporal bone and some fluid in mastoid cells

An MRA and MRI was performed postoperatively (after 1 month) which revealed no evidence of disease in the vascular system, the presence of chronic inflammatory lesions in petrous part of temporal bone and some fluid in mastoid cells, that can be justified from the surgery and it is expected to be absorbed (Figure 5). At 1-year follow-up patient remains disease free.

## Discussion

Glomus tumor or chemodectoma or paraganglioma, constitute the most common primary neoplasm of the middle ear, and the second most common tumor of the temporal bone, called glomus tympanicum (4). There are two types of paragangliomas, sympathetic and parasympathetic, second type is most often found in the head and neck (5). It usually does not release catecholamines, so it causes hormone-related symptoms or signs of a problem rarely (5). Glomus tympanicum arises from the paraganglia of the middle ear. Histologically, it is similar to carotid body tumors which constitute the most common type of paragangliomas (6). 

Around 5% to 10% of these tumors secrete catecholamines (epinephrine, norepinephrine, dopamine) causing hypertension, headaches, flushing and cardiac arrhythmias (5). 

The vast majority of these tumors are benign and slow growing and only rarely they present aggressive behavior (1,5). These tumors are more common in women (7). They are presented with pulsatile tinnitus and conductive hearing loss (1,8). 

The differential diagnosis includes cholesteatoma, tympanic paraganglioma, glomus jugulare, high riding jugular bulb, ectopic position of the jugular bulb and ectopic carotid artery. The challenge in diagnosis was to decide if it was a vascular tumor or not. If it was a vascular tumor an embolism before surgery should had been scheduled. Considering that patient reported no bleedings from the ear and in light of radiology report, a misdiagnosis of cholesteatoma was made. However, despite the fact that first diagnosis was wrong, our team managed the bleeding during the operation and removed the whole mass safely. 

This makes our case rare and interesting. Diagnosis could be more accurate if an intravascular agent had been administrated in the first CT scan. It had not happened because of lack of patient’s recent urine and creatinine results. Facial nerve paralysis may be caused as a result of tumor expansion in the mastoid cavity, just as in our case and we used intravenous steroids to prevent permanent complications. Moreover, considering the possible complications, we administrated a course of antibiotics after the first examination in order to resolve otorrhea and prevent further spreading of inflammation in temporal bone or intracranial. The most common used imaging studies to assess a paraganglioma are: CT and magnetic resonance imaging (MRI) of the temporal bone and skull base (5,9). Additionally, our case report provides detailed information regarding the management of glomus tympanicum and highlights the possibility of misdiagnosis in such cases. Furthermore, it is emphasized the importance of immediate administration of steroids in facial nerve palsy and the significance of canal wall down mastoidectomy in big tumor ‘s exclusion, which is also supported in literature (10,11). Generally, it is recommended canal wall down technique to be done if the mastoid is involved, the external auditory canal was eroded by the tumor or there is extension of the tumor into anterior epitympanum (11).

## Conclusions

It is of high importance that every otolaryngologist should recognize such a tumor and be aware of a possible disastrous bleeding event in case of probing in clinic. An imaging technique with intravascular contrast media would be necessary and helpful for an accurate diagnosis in future similar cases. 

The treatment is individualized since for older patients with poor physical condition radiotherapy is the treatment of choice, while younger ones are candidates for surgical removal after angiography and embolism (1,12-14). 

Physicians should also be open to other modalities of treatment, including use of the gamma knife. Method of consent was verbal for this article.
